# Brace treatment in juvenile idiopathic scoliosis: a prospective study in accordance with the SRS criteria for bracing studies - SOSORT award 2013 winner

**DOI:** 10.1186/1748-7161-9-3

**Published:** 2014-04-23

**Authors:** Angelo G Aulisa, Vincenzo Guzzanti, Emanuele Marzetti, Marco Giordano, Francesco Falciglia, Lorenzo Aulisa

**Affiliations:** 1 U.O.C. of Orthopedics and Traumatology, Children’s Hospital Bambino Gesù, Institute of Scientific Research, P.zza S. Onofrio 4, 00165 Rome, Italy; 2 University of Cassino, Cassino, FR 03043, Italy; 3 Department of Orthopedics, University Hospital "Agostino Gemelli", Catholic University of the Sacred Heart School of Medicine, Rome 00168, Italy

**Keywords:** Juvenile idiopathic scoliosis, Scoliosis research society criteria (SRS), PASB brace, Lyon brace, SOSORT guidelines, Conservative treatment

## Abstract

**Background:**

The Juvenile idiopathic scoliosis by age of onset, severity and evolutivity is source of great doubts concerning the purpose and use of conservative treatment. The different clinical experiences leave unsolved the question that arises in applying a conservative treatment when the patients are effectively forward a long growing period, in scoliosis characterized by inevitable evolutivity. The purpose of the present prospective study was to determine the effectiveness of conservative treatment in Juvenile idiopathic scoliosis.

**Methods:**

From 1238 patients treated for idiopathic scoliosis between 1995 and 2012 fulfill the inclusion criteria 163 patients treated with PASB, Lyon brace and Milwaukee. Of these, 113 patients had a definite outcome, 27 have abandoned treatment e 23 are still in treatment. The minimum follow-up was 24 months. Radiographs were used to estimate the curve magnitude (CM) and the torsion of the apical vertebra (TA) at 5 time points: beginning (t1), 6 months after the beginning (t2), intermediate time between t1 and t4 (t3), end of weaning (t4), 2-years minimum follow-up (t5). Three outcomes were distinguished in agreement with SRS criteria: correction, stabilization and progression.

**Results:**

The results from our study showed that of the 113 patients with a definite outcome CM mean value was 29.6 ± 7.5 SD at t1 and 16.9 ± 11.1 SD at t5. TA was 13.5 ± 5.4 SD at t1 and 8.5 ± 5.6 at t5. The variations between CM t5-t1 and TA t5-t1 were statistically significantly different. Curve correction was accomplished in 88 patients (77.8%), stabilization was obtained in 18 patients (15.9%). 7 patients (6.19%) have a progression and 4 of these were recommended for surgery. Of 26 patients who abandoned the treatment, at the time of abandonment (12.5 age) have achieved curve correction in 19 cases (70.0%), stabilization in 5 cases (19%) and progression in 3 cases (11%). Of these patients, reviewed at the end of growing, four have been operated on.

**Conclusions:**

Our study confirmed that conservative treatment with brace is highly effective in treating juvenile idiopathic scoliosis, in particular most patients reaching a complete curve correction and only 4.9% of patients need surgery.

## Background

The classification of idiopathic scoliosis is based on chronologic criteria related to the age of curve presentation (Terminology Committee of Scoliosis Research Society). The first to divide the idiopathic scoliosis in juvenile (presentation up to 10 years), and adolescent (presentation past 10 years) was Ponseti in 1950 [1]. In 1954, James proposed the distinction of idiopathic scoliosis in infantile, juvenile and adolescent: "Idiopathic scoliosis begins at all ages of childhood but there are three peak periods of onset - under the age of three, from five to eight and from ten until the end of growth. For convenience I have called these age groups, infantile, juvenile and adolescent" [2]. Successively, in 1970, James proposed a refinement of the age range: "Some Authors have regarded infantile idiopathic scoliosis as occurring within the first two years of life. I have used the end of the third year to define a curve as being of infantile onset. I have also used the terms juvenile for an onset of scoliosis in the years 4–9 and adolescent scoliosis for the onset of curves between the age of ten and the end of growth." [3]. This classification was subsequently confirmed from Goldstein in 1973 [4]. However, in 2010, the Scoliosis Research Society (SRS) defined juvenile idiopathic scoliosis (JIS) as a scoliosis that is first diagnosed between the ages of 4 and 10 [5].

The incidence of juvenile scoliosis has been reported to be comprised between 7 and 17% [1,2,5-10]. The discrepancy in incidence data may be attributed to several factors, such as the heterogeneity of the examiners, the different parameters of identification and the size of the sample analyzed. In our series, among 1,238 cases of idiopathic scoliosis who had undergone conservative treatment, JIS was present in 163 subjects (prevalence: 13%).

The natural course of JIS is much more aggressive than that of adolescent idiopathic scoliosis. Cotrel [11] reported that JIS at the end of growth showed a curve between 50 and 100° Cobb in 41% of patients, and greater than 100° in 45% of cases. Duval-Beaupère observed that patients with juvenile scoliosis presented an annual increase in curve magnitude from 4° to 7° Cobb until the prepubertal period, with an average of 23° Cobb increase in the following period (puberty - skeletal maturity) [12]. Mannherz [13] found that juvenile curves progress in 70-95% of patients and about half of this patients will need surgery. Other authors confirmed that juvenile curves of 30° Cobb or greater tend to worsen in the absence of treatment, nearly 95% require surgery [14,15].

In the last years, evidence has shown that bracing is the most effective non-surgical treatment; however, data are limited to adolescent idiopathic scoliosis [16-25]. In the case of JIS, the opportunity and outcomes of conservative treatment are still a matter of debate [26,27]. In most studies, observation is the first treatment in all cases with mild curves (<20°), but treatment should be considered earlier if the in patients showing curve progression and/or with family history of scoliosis. In patients with curves over 25°, treatment is usually indicated due to the high probability of progression. Discrepancies in clinical experiences leave unsolved the question as to whether conservative treatment need to be implemented in juvenile scoliosis [28,29]. Actually, several authors believe that conservative treatment does not represent a truly effective option, but only a procrastination of surgery, with the aim of limiting the evolution of the curves and waiting for the right age to intervene [30,31]. Nevertheless, some authors have shown a reduction of the incidence of surgery [32,33].

Based on these premises, the present study was undertaken to determine the effects of conservative treatment in JIS.

## Methods

### Patients

This is a prospective study based on ongoing database including 1238 patients treated for idiopathic scoliosis between 1990 and 2012. Inclusion criteria were: age at the beginning of treatment of 4 to 10 years and curve magnitude (C_M_) 20°-40° Cobb. Curves between 20° and 25° Cobb degrees were included only in the presence of documented curve progression. Curve progression was assessed on two consecutive X-rays taken at 6-month interval and was defined as an increase greater than 5° in C_M_ (Cobb’s method) [27]. The minimum duration of follow-up was 24 months after the end of treatment.

One-hundred sixty-three patients met the inclusion criteria. Of these, 113 patients had definite outcome, 27 abandoned the treatment, and 23 are currently under treatment.

### Bracing

Patients with thoraco-lumbar and lumbar curves were prescribed with Progressive Action Short Brace (PASB), while Milwaukee or Lyon brace were prescribed in those with thoracic or double curves. All patients were prescribed with full-time bracing (i.e., max 22 hours daily, min 18 hours daily). Daily hours of bracing were defined for each patient according to clinical needs and acceptance. In order to maximize the adherence to treatment, patients were always followed by the same doctor [34]. Furthermore, controls were performed every 2 months until Risser 3, and every 3 months thereafter. Frequent checks allowed to verify and implement compliance establishing an open and friendly relationship with the patients. Close checks were also performed to maximize bracing effectiveness over the time.

Weaning was started when ring-apophysis fusion was seen to begin on a latero-lateral (LL) radiograph view [35], which corresponds to a Risser sign 4 or 5 on an antero-posterior (AP) standing radiograph view. Weaning consisted of 2 to 4 hours bracing reduction at 2-month intervals. The curve response to progressive part-time bracing was evaluated on an AP view standing radiograph after the patient had been without bracing for 5 hours. Out-of-brace hours were not reduced and in some cases increased if the curve was not stable.

### Endpoints

For the present study, only the X-ray performed at conventional times were considered: beginning of treatment (t_1_), 4–6 months after the beginning of treatment (t_2_), intermediate time between t_1_ and t_4_ (t_3_), end of weaning (t_4_), 2-year minimum follow-up from t_4_ (t_5_). For each patient, AP and LL view standing X-rays of the whole spine were performed. X-rays before treatment (t_1_) as well as those at t_4_ and t_5_ were taken while out of brace. All other radiographic controls were performed with the patient wearing the brace, in order to assess the corrective action of bracing. The first X-ray was obtained at 4–6 months from the beginning of treatment. All other controls were performed once a year. All radiographs were taken at our Institute, at 2-meter distance, on a 36 × 91 cm film. The AP view was used to determine the patient’s skeletal age (Risser’s sign) and to obtain the C_M_ and torsion of the apical vertebra (T_A_: Perdriolle’s method). Measurements were obtained by two independent observers. The end-vertebrae were pre-selected to reduce inter-observer error [27]. Curves were classified according to SRS into thoracic, thoracolumbar, lumbar, and double major. As recommended by the SRS Committee on Bracing and Non-operative Management, outcomes were classified as follows: (1) correction (percentage of patients with ≤ 5° curve progression), (2) stabilization (percentage of patients with > -5 and < 5°changes in C_M_), (3) progression (percentage of patients with ≥ 5° progression at maturity), and (4) percentage of patients with curves exceeding 45° at maturity and those who were recommended for or had undergone surgery.

### Statistical analysis

Statistical analysis was performed using the SPSS v.13.0 software (SPSS Inc.; Chicago, IL). For all variables, normality of data was ascertained by the Kolmogorov-Smirnov’s test. Results were analyzed in relation to C_M_ t_5_-t_1_ at follow-up, assuming that C_M_ t_5_-t_1_ was not within the Cobb’s method ± 5 range error [27]. Changes in C_M_ and T_A_ over time from t_1_ through t_5_ were assessed via one-way analysis of variance (ANOVA) for repeated measures. Tukey’s post-test was applied when needed. The model was adjusted for age, gender, type of curve, and type of bracing. All analyses were performed according to the intention-to-treat principle. Missing data at follow-up were managed according to the Last Observation Carried Forward (LOCF) method. All tests were two-sided, with significance set at p < 0.05. Results are presented as mean ± standard deviation (SD).

## Results

### Analyses of patients with a definite outcome

A definite outcome was recorded for 113 patients, 104 females (93%) and 9 males (7%), mean age 8.1 ± 1.2 years and 16.3 ± 1.9 years at t1 and t4, respectively. The mean duration of treatment was 84.2 ± 16.7 months, with an average length of follow-up of 56.9 ± 27.0 months.

Curve type distribution was as follows: thoracic (n = 32; 28.3%), thoraco-lumbar (n = 30; 26.5%), lumbar (n = 23; 20.4%), and double (n = 28; 24.8%).

**Figure 1:**
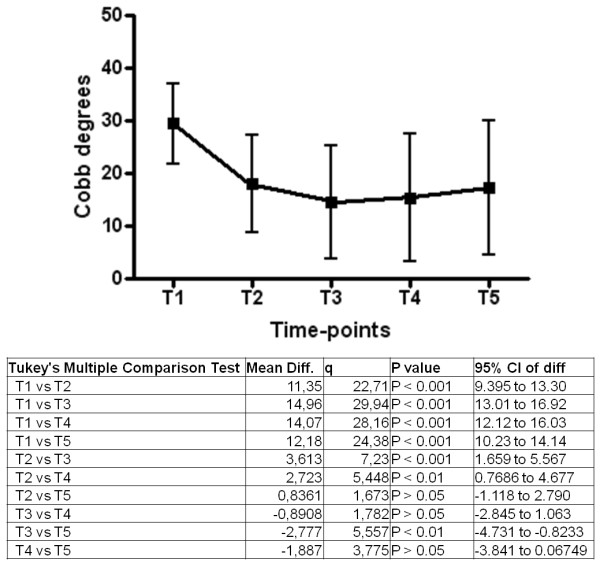
Changes in CM over time in the whole study sample (n = 113).

**Figure 2:**
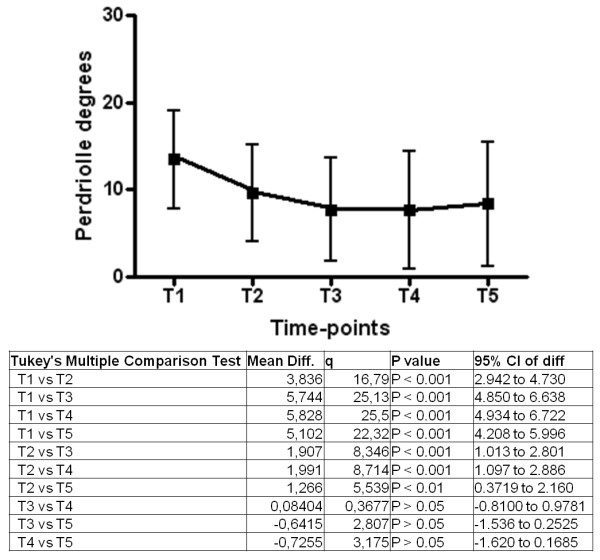
Changes in TA over time in the whole study sample (n = 113).

Changes in C_M_ over time were statistically significant (p for trend < 0.0001) [Figure 1], with a mean value of 29.6 ± 7.5° Cobb at t_1_ and 16.9 ± 11.1° Cobb at t_5_. A similar pattern was observed for T_A_ (p for trend < 0.0001) [Figure 2], with a mean value of 13.5 ± 5.4° Perdriolle at t_1_ and 8.5 ± 5.6° Perdriolle at t_5_.

**Figure 3:**
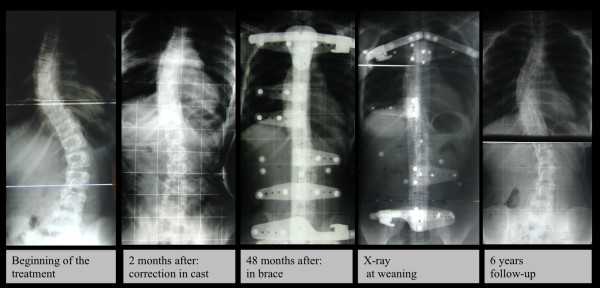
This is a case with a thoracic curve treated with Lyon Brace; Cobb degrees value was 40°at beginning of treatment and 24° at 6 years of follow-up.

**Figure 4:**
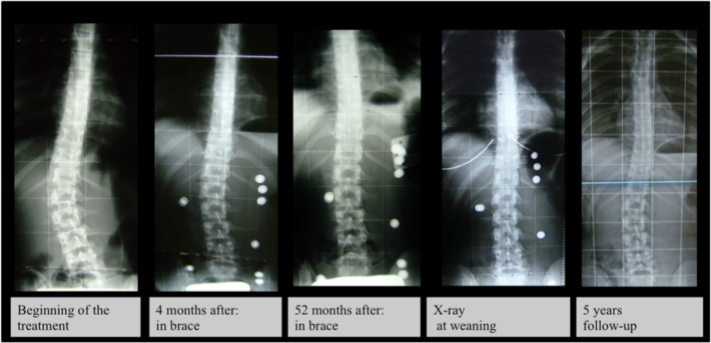
This is a case with a lumbar curve treated with PASB Brace; Cobb degrees value was 29°at beginning of treatment and 8° at 5 years of follow-up.

Overall, 88 patients (77.8%) obtained a curve correction, while stabilization was achieved in 18 cases (15.9%). Curve progression was experienced by 7 patients (6.2%), 4 of whom were subsequently recommended for surgery. In patients with C_M_ < 30° at t_1_, the average reduction was -12.6° Cobb, while in cases with C_M_ ≥ 30 at t_1_, the mean correction was 11.8° Cobb. As for the curve type, patients with lumbar curves obtained an average correction of 14.7° Cobb [Figure 3], whereas a 8.0° Cobb decrease was observed in cases with thoraco-lumbar curves and 10.0° Cobb in those with thoracic curves [Figure 4]. Finally, patients with double curves experienced an average 12.5° Cobb reduction.

Treatment outcomes according to C_M_ at baseline and curve type are shown in Table 1.

**Table 1:** Treatment outcome according to curve severity at baseline and type of curve

	**Curve correction (n, %)**	**Curve stabilization (n, %)**	**Curve progression (n, %)**	**Surgery referral (n, %)**
**Curve severity at t**_**1**_				
< 30° Cobb (n = 59)	49(83%)	7	2	1(1.6%)
≥ 30° Cobb (n = 54)	39(72.2%)	11	5	3(5.5%)
**Curve type**				
Lumbar (n = 23)	23 (100%)	0 (--)	0 (--)	0 (--)
Thoraco-lumbar (n = 30)	26 (86.7%)	4 (13.3%)	0 (--)	0 (--)
Thoracic (n = 32)	21 (65.6%)	7 (21.9%)	4 (12.5%)	3 (9.4%)
Double (n = 28)	18	7	3	1 (3.6%)

### Analyses of patients who abandoned the treatment

Twenty-seven patients abandoned the treatment, 26 females (96%) and 1 male (4%), mean age: 8.0 years at t_1_ and 12.5 years at the time of discontinuation.

C_M_ was 27.9 ± 6.9° Cobb at t_1_ and 17.3 ± 8.4° Cobb at the time of bracing discontinuation, with a mean correction of -10.6°. Curve correction was observed in 19 cases (70%), stabilization in 5 patients (19%), and progression in 3 patients (11%). Among 18 patients who were recontacted at the end of growth, 14 showed a progression of 12,7° Cobb and 4 had undergone surgery.

## Discussion

The main objective of the present study was determine the effects of conservative treatment in JIS through a prospective approach. The study was conducted according to the SRS Committee criteria and the guidelines on standard of management of idiopathic scoliosis with corrective braces in everyday clinics and in clinical research proposed by the Society on Scoliosis Orthopaedic and Rehabilitation Treatment (SOSORT) [24,34]. Analyses of our case series revealed that the large majority of patients with a definite outcome (82.5%) obtained a curve correction after brace treatment, whereas a curve stabilization was accomplished in 15.9% of cases. Only 4 patients (3.5%) were subsequently recommended for surgery during the follow-up. Taken as a whole, the current findings together with our previous observations suggest that the brace treatment is an effective option in JIS.

In the literature there are very few publications that have evaluated the effects of conservative treatment in JIS patients taking the outcome into account. The success rate of orthotic programs in the management of JIS is variable among the different authors, with conservative treatment mainly centered at slowing/stopping the progression of the curve and avoiding or delaying spine fusion. Kahanovitz [36] reported an excellent prognosis with part-time bracing for smaller curves and a poor prognosis in patients with greater Cobb angles, all of whom eventually needed surgery. Tolo and Gillespie [37] found that only 27.2% (16/59) of their patients treated with the Milwaukee brace needed surgery. Similar results were reported by Dabney and Browen [38], with 33% of surgery recommendations. Other authors have reported much higher percentages of patients who needed surgery despite bracing. For instance, Figueiredo and James [39] reported a 62% incidence of surgery in patients treated with a modified Milwaukee brace, Mannherz [13] reported 80%, and McMaster 87% [10]. In a recent paper, Jarvis [32] highlighted the difficult in comparing the results of the various studies because they involve patients with different characteristics, non standard indications for surgery, which varies from 19% to 87%, and outcome analysis. Moreover, he showed that patients treated with part-time Charleston bracing obtained correction in 52% of cases and underwent surgery in 30%.

To date, the only prospective study adopting the SRS criteria for outcome evaluation in juvenile scoliosis has been performed in patients treated with Dynamic SpineCor bracing [33]. Fifty-seven% of cases reached a curve correction or stabilization. However, 37% of patients needed surgical fusion while receiving treatment (26.3% with curves < 25° and 51.8% with curves > 25°).

Comparing our series to the findings reported above, we showed 75% of correction and only 5% of surgery in a larger sample of patients and with different braces. Furthermore, significant correction was detected both for C_M_ and T_A_, demonstrating the efficacy of treatment on both parameters.

The greatest correction was observed in cases treated with PASB (lumbar and thoraco-lumbar curves), with none of the patients showing curve progression (>5°) at follow-up. In addition, correction was achieved early during treatment. This might have occurred because in the initial phase bracing acts mostly on the elastic component of the curve, leading to an early, substantial correction. However, derotation and vertebral remodeling proceed during the entire course of treatment, ensuring further curve correction and its maintenance over time.

With regard to curve severity, it is worth noting that patients with curves under 30° obtained a correction in 83% of cases (incidence of surgery: 1.6%), while curves over 30° reached a correction in 72.2% of cases, with surgery recommended in 5.5% of patients. These results cannot be explained only by mechanical aspects. Indeed, the response of the scoliotic spine to the actions exerted by the orthosis is determined by two factors: the ability to remodel the vertebrae (in accordance with the law of Hueter-Volkman) and the suitability of visco-elastic structures to respond adequately to the action of bracing. Any mechanical strain appears inadequate to promote the remodeling process without an adequate visco-elastic response of the structures involved. Therefore, the discs included in the scoliotic curve must be able to work in the field of linear elasticity. The state of disc’s hysteresis, in fact, would make it unable to transmit effective actions for recovery of the deformity [40,41]. Hence, the greater the rotation of the curve, the less the capacity of its correction. Therefore the early diagnosis of scoliosis is very important and to facilitate early administration of conservative treatments we can use school screening that is predictive and sensitive tool with a low referral rate [42,43].

About the patients who abandoned the treatment the results showed a progression of curve, at the time of discontinuation, only in the 11% of cases. Therefore, were not the results to send away the patient but, probably, the trouble of a long term treatment. In particular the failure rate of treatment including the dropouts is 24% but the surgical rate is 12%.

## Conclusions

The results obtained in this prospective study clearly show that brace treatment (PASB, Lyon and Milwaukee) can alter the natural history of JIS and that the correction appears to be maintained over the long term. The treatment appears to be more effective with curves under 30° (incidence of surgery: 1.6%) than curves over 30° (incidence of surgery: 5.5%) but compared to the natural history of disease both are better. Moreover these results confirm that the adoption of conservative approaches based on the SOSORT and SRS guidelines produce better results. Nonetheless, these results highlight the necessity for new studies to evaluate the effectiveness of conservative treatment in Juvenile idiopathic scoliosis in curves over 40 degrees.

## Abbreviations

JIS: Juvenile idiopathic scoliosis; SRS: Scoliosis Research Society; PASB: Progressive action short brace; CM: Curve magnitude; LL: Latero-lateral; AP: Antero-posterior; T_A_: Torsion of the apical vertebra.

## Competing interests

The authors declare that they have no competing interests.

## Authors’ contributions

All authors contributed equally to this work, all authors read and approved the final manuscript.
